# Chronic spinal cord injury treated with transplanted autologous bone marrow-derived mesenchymal stem cells tracked by magnetic resonance imaging: a case report

**DOI:** 10.1186/s13256-015-0535-6

**Published:** 2015-04-09

**Authors:** Areesak Chotivichit, Monchai Ruangchainikom, Pipat Chiewvit, Adisak Wongkajornsilp, Kittipong Sujirattanawimol

**Affiliations:** 1 Department of Orthopaedic Surgery, Faculty of Medicine Siriraj Hospital, Mahidol University, 2 Prannok Rd, Bangkoknoi, Bangkok 10700 Thailand; 2 Department of Radiology, Faculty of Medicine Siriraj Hospital, Mahidol University, 2 Prannok Rd, Bangkoknoi, Bangkok 10700 Thailand; 3 Department of Pharmacology, Faculty of Medicine Siriraj Hospital, Mahidol University, 2 Prannok Rd, Bangkoknoi, Bangkok 10700 Thailand; 4 Department of Anesthesiology, Faculty of Medicine Siriraj Hospital, Mahidol University, 2 Prannok Rd, Bangkoknoi, Bangkok 10700 Thailand

**Keywords:** Spinal cord injury, Mesenchymal stem cells, Superparamagnetic iron oxide, Magnetic resonance imaging, Tracking

## Abstract

**Introduction:**

Intrathecal transplantation is a minimally invasive method for the delivery of stem cells, however, whether the cells migrate from the lumbar to the injured cervical spinal cord has not been proved in humans. We describe an attempt to track bone marrow-derived mesenchymal stem cells in a patient with a chronic cervical spinal cord injury.

**Case presentation:**

A 33-year-old Thai man who sustained an incomplete spinal cord injury from the atlanto-axial subluxation was enrolled into a pilot study aiming to track bone marrow-derived mesenchymal stem cells, labeled with superparamagnetic iron oxide nanoparticles, from intrathecal transplantation in chronic cervical spinal cord injury. He had been dependent on respiratory support since 2005. There had been no improvement in his neurological function for the past 54 months. Bone marrow-derived mesenchymal stem cells were retrieved from his iliac crest and repopulated to the target number. One half of the total cells were labeled with superparamagnetic iron oxide nanoparticles before transplantation to the intrathecal space between L4 and L5. Magnetic resonance imaging studies were performed immediately after the transplantation and at 48 hours, two weeks, one month and seven months after the transplantation. His magnetic resonance imaging scan performed immediately after the transplantation showed hyposignal intensity of paramagnetic substance tagged stem cells in the subarachnoid space at the lumbar spine area. This phenomenon was observed at the surface around his cervical spinal cord at 48 hours. A focal hyposignal intensity of tagged bone marrow-derived stem cells was detected at his cervical spinal cord with magnetic resonance imaging at 48 hours, which faded after two weeks, and then disappeared after one month. No clinical improvement of the neurological function had occurred at the end of this study. However, at 48 hours after the transplantation, he presented with a fever, headache, myalgia and worsening of his motor function (by one grade of all key muscles by the American Spinal Injury Association impairment scale), which lasted for 48 hours.

**Conclusion:**

Intrathecal injection of bone marrow-derived stem cells at the lumbar spine level could deliver the cells to the injured cervical spinal cord. Transient complications should be observed closely in the first 48 hours after transplantation. Further study should be carried out to evaluate the result of the treatment.

## Introduction

Spinal cord injury usually results in long-lasting disability as there are limited treatments for improving neurological condition. Finding a new specific treatment to treat such injury is a major clinical challenge, however, stem cell transplantation is a possible way of repairing the injured spinal cord [1,2]. Autologous bone marrow-derived mesenchymal stem cells (BMSCs) are one type of stem cells that have been well-reported on in spinal cord injury studies [3,4].

Intrathecal transplantation is an attractive approach for BMSC transplantation because it requires a simple technique and less invasive approach. Intrathecal BMSC transplantation allows for the efficient delivery of cells to the injured spinal cord [5-9]. Intrathecal transplantation can deliver BMSCs to the spinal cord through the blood–brain barrier in rat models; moreover, BMSCs infused into cerebrospinal fluid promote functional recovery of the injured spinal cord, with reduced cavity formation [8,10,11]. Nevertheless, intrathecal transplantation is not a direct transplant into the injured site of the spinal cord; the migration of intrathecally transplanted cells at the lumbar level to higher injured areas such as the cervical spinal cord is questionable in humans, especially in cases of chronic injured spinal cord. Cell tracking technology would help to gain more information regarding cell migration in intrathecal transplantation.

Cell labeling with superparamagnetic iron oxide nanoparticles (SPIONs) is an interesting choice for stem cell tracking in cases of spinal cord injury [12,13]. It is an intracellular labeling that provides high specificity for tracking living cells. Labeling cells with SPIONs is simple to prepare and, in low doses, does not affect the proliferation and differentiation of cells [14-16]. Moreover, cells labeled with SPIONs are detectable using magnetic resonance imaging (MRI), which is useful for clinical evaluation.

We describe the case of a patient with a chronic cervical spinal cord injury who was enrolled in a study which attempted to track BMSCs labeled with SPIONS after intrathecal transplantation. In addition, the clinical outcomes and safety of using intrathecal SPION-labeled BMSC transplantation was evaluated.

## Case presentation

We conducted a pilot study for patients with a complete or incomplete spinal cord injury at the cervical spine level, occurring more than two years previously. Our patient was recruited from the spinal unit at Siriraj Hospital (Bangkok, Thailand). The protocol for this study was reviewed and approved by the local ethics committee (Ethics Committee for Research in Humans, Faculty of Medicine Siriraj Hospital, Mahidol University (approval number: Si084/2009). This research was registered With Thai Clinical Trials Registry (www.clinicaltrials.in.th); the register number is 20140611001). Our patient was the sole patient to enroll in this pilot study.

**Figure 1:**
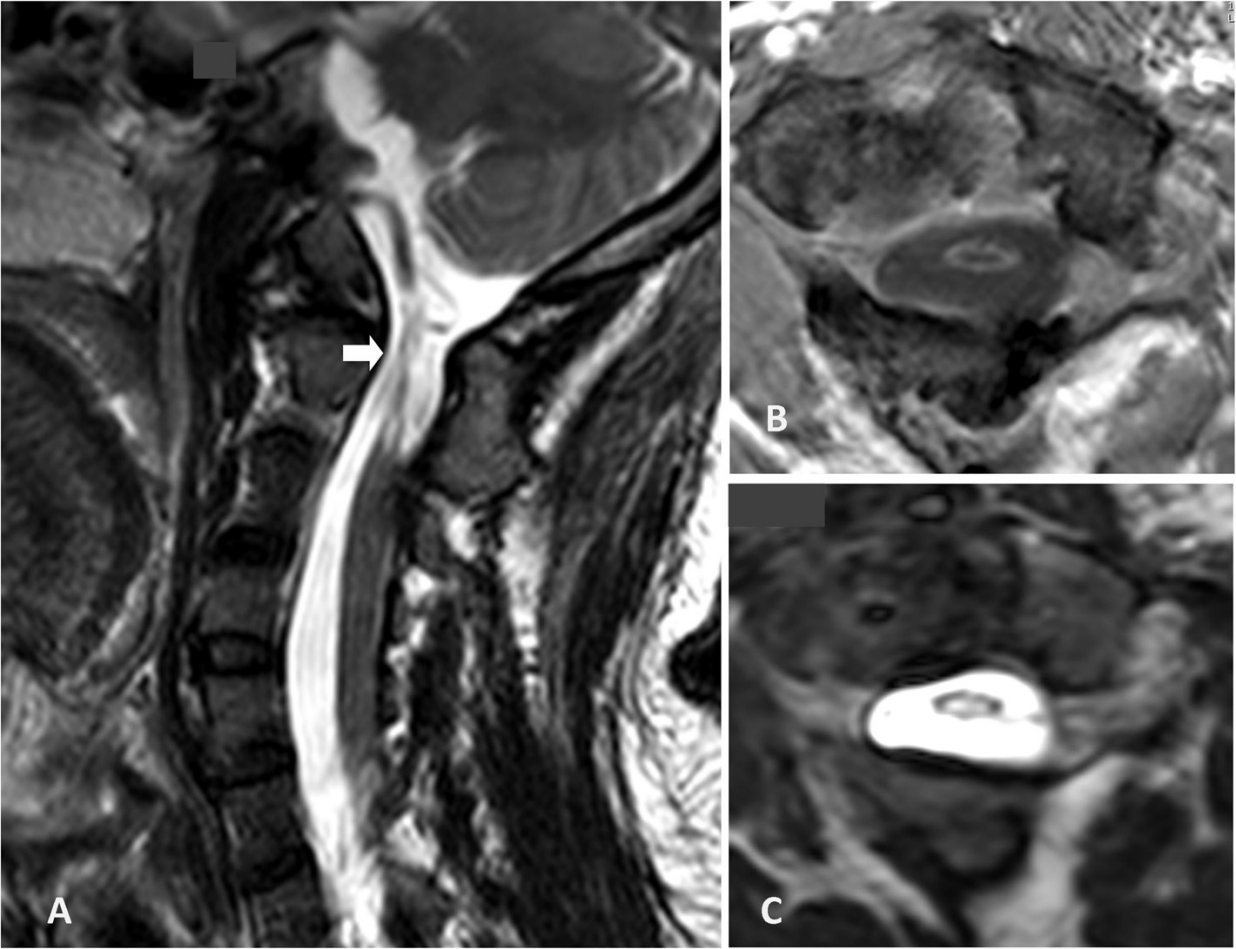
**Cervical spinal cord magnetic resonance imaging scan prior to stem cell transplantation.** Sagittal and axial T2-weighting **(A, C)** and axial T1-weighting **(B)** scans revealed the small diameter of the cervical spinal cord at the C1-C2 level, which demonstrated the atrophic change of the spinal cord (arrowhead).

Our patient was a 33-year-old Thai man who sustained an incomplete spinal cord injury, quadriparesis, from atlantoaxial subluxation. MRI showed that his spinal cord was compressed from the posterior arch of the atlas. He was reduced with traction and received atlantoaxial fusion with wiring and post-operative cervical spine maintenance using Halo vest immobilization in May, 2005. Neurological deficits had partially improved from the operation. At four years and six months later, his neurological condition was still quadriparesis, and he was still dependent on respiratory support via a tracheostomy. His respiratory functions and motor power functions did not improved any further, despite intensive rehabilitation treatment. His sensory function was mainly preserved, however, neuropathic pain was predominant over his entire body, especially on his left side. His cervical spine was evaluated with a computed tomography (CT) scan for bony fusion, and his atlantoaxial joint had already fused. His spinal cord lesion was evaluated with 3.0 Tesla MRI in 2009. His cervical spinal cord at the C1-C2 level was small in diameter, representative of atrophic myelomalacia change of his cervical spinal cord from level C1 to the intervertebral disc of level C2-C3 (Figure 1).

We discussed this study with our patient and his family thoroughly to avoid any ethical issues. He and his family made the decision and provided signed informed consent to be enrolled in this study.

A total of 200mL of bone marrow aspiration was harvested from his iliac crest. Mononuclear cells were separated with Ficoll-Hypaque density gradient centrifugation (IsoPrep®, Robbins Scientific, Sunnyvale, CA, USA). The mononuclear cells were cultured with Dulbecco’s modified eagle’s medium (DMEM) (Gibco®, Life Technologies^TM^, NY, USA) with 10% fetal bovine serum (Biochrom, Berlin, Germany). The culture was maintained at 37°C in a humidified atmosphere of 5% carbon dioxide. Culture medium was replaced every four days. The cells were maintained in the primary culture for four weeks in order to increase the number of cells. The target number of culture stem cells was 30×10^6^ cells. A sample of the BMSCs was cultured to test for bacteria and fungus; the results were negative. Half of the BMSCs (15×10^6^ cells) were separated for labeling with SPIONs. The superparamagnetic iron oxide agent (Resovist®, Schering, Berlin, Germany) was administered to the culture medium at a concentration of 100μg/mL and the transfection agent (protamine sulphate (Leo Pharmaceutical, Ballerup, Denmark)) was also administered to the culture medium at a concentration of 4μg/mL, which was then mixed for approximately 10 minutes. Following mixing, 12.5mL of the labeling medium was added to an 80% confluent BMSC monolayer in a 150cm^2^ cell culture flask. After two hours of incubation, an equal volume of complete DMEM was added to the cultures for a final SPION concentration of 50μg/mL. Cell cultures were incubated overnight. Samples were washed with phosphate-buffered saline (PBS) containing heparin (Leo Pharmaceutical, Ballerup, Denmark) at a concentration of 10 US Pharmacopeia (USP) units/mL. The BMSCs labeled with SPIONs and the unlabeled BMSCs were mixed and re-suspended in normal saline. Intrathecal BMSC transplantation was performed at the lumbar spine. Then, an immediate MRI scan was carried out to confirm that the BMSCs were injected into the arachnoid space. After intrathecal BMSC transplantation, he was set in the Trendelenburg position for 24 hours.

We performed MRI scans using a 3.0 Tesla Philips Achieva MRI scanner (Philips Medical Systems, Best, Netherlands) with a combination of two different phase array coils: a sensitivity encoding (SENSE) spine coil and a SENSE- neurovascular coil, a combination in terms of dual-coil imaging. We examined the T1-weighted sagittal spin echo images (the repetition time (TR)=584 milliseconds; the echo time (TE)=6.6 milliseconds; slice thickness=2.0mm; field of view=25×25cm; matrix=288×230; and number of excitations=8 (NEX)) and the T2-weighted sagittal fast spin echo images (TR= 4017 milliseconds; TE=100 millisecond repetition time; slice thickness=2.0mm; field of view=25×25cm; matrix=352×308; and NEX=8) of each patient. T2-star echo images (TR=14.8 milliseconds; TE=multiechoes=3.57/4.93/6.29/7.65/9.0/10.36/11.72/13.08; slice thickness=3.0mm; field of view=15×15cm; matrix=152×144; and NEX=4). The MRI scan was performed immediately after stem cell transplantation. In addition, the same protocol of whole spinal axis MRI was performed at two days, two weeks, one month and seven months after the transplant.

At 48 hours after transplantation, he had a fever (temperature: 38 to 38.5°C), headache and myalgia. There were transient neurological deficits. All key muscles by the American Spinal Injury Association (ASIA) impairment scale decreased by one grade of all key muscles by ASIA Impairment scale, followed by loss of penile erection the next morning; however, his sensory by ASIA impairment scale was still intact. His physical examination revealed no stiff neck and his CBC revealed mild leukocytosis. Hemocultures were taken and the results were negative. An immediate MRI scan was performed and did not reveal any significant change. He was treated with 10mg intravenous dexamethasone every six hours for two days. His neurological status had improved to pre-transplant status within 12 hours after the dexamethasone treatment. For the first six months after transplantation there was no change in both neurological status and respiratory function. At 12 months after the transplantation, his neuropathic pain increased on both sides of his body. At present, his left side is still experiencing more pain than his right side and his respiratory function has not significantly changed, remaining respirator-dependent (Table 1).

**Table 1:** **Summary of clinical data from pre-operative time to 12 months post-operative time**

	**Preoperative data**	**Postoperative data**		
		**2 days**	**6 months**	**12 months**
ASIA impairment scale	B	B	B	B
Respirator	dependent	dependent	dependent	dependent
Motor score				
Upper limbs motor score	18	10	18	18
Lower limbs motor score	4	2	4	4
Sensory score				
Light touch score	109	109	109	109
Pin prick score	108	108	108	108
Neuropathic pain	+	+	+	++

**Figure 2:**
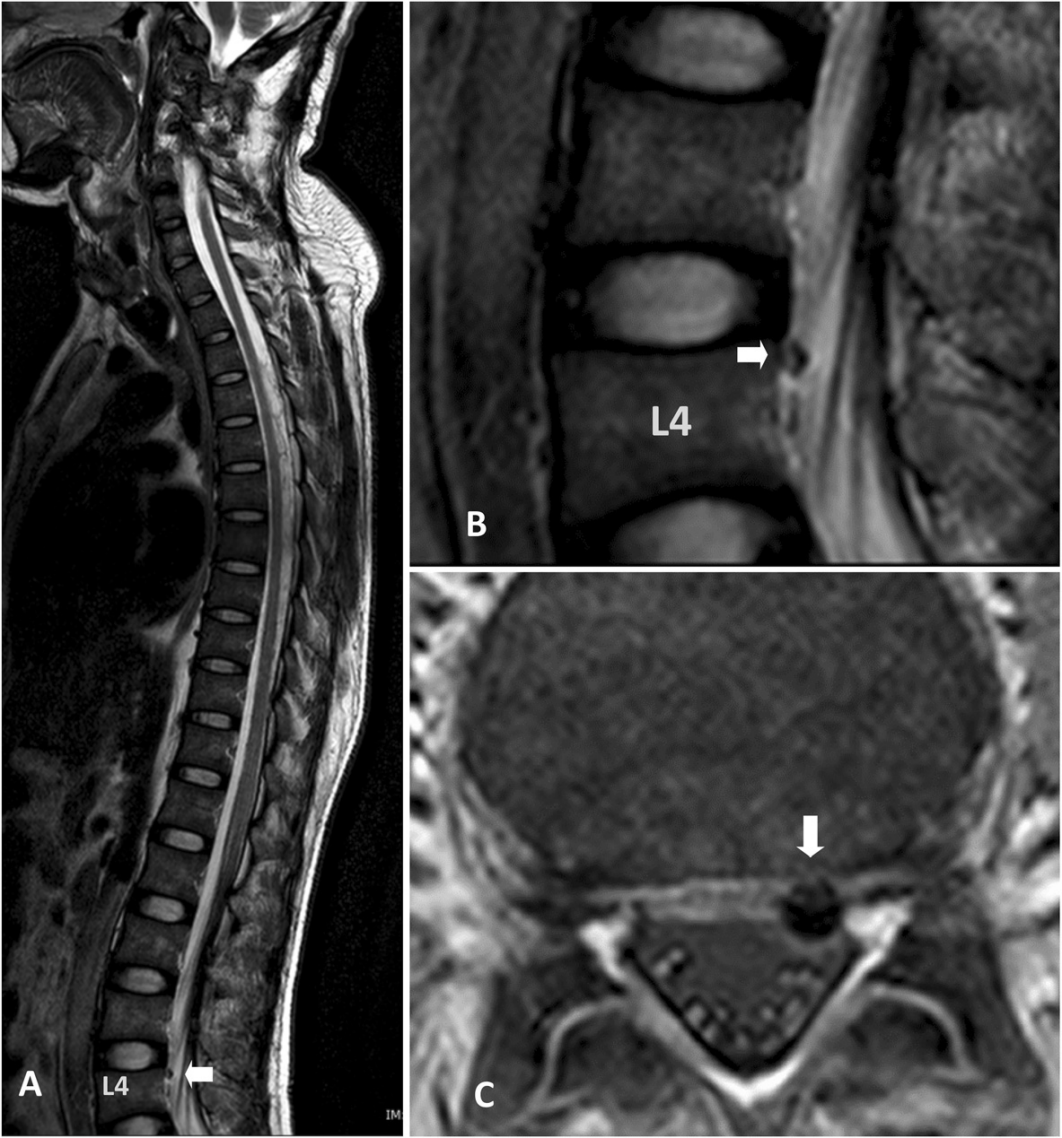
**Magnetic resonance imaging scan of the cervical spine taken one day after stem cell transplantation via lumbar puncture technique.** A sagittal T2-weighted scan of his whole spine **(A, B)** and an axial T2-star scan of his lumbar spine **(C)** shows hyposignal intensity of paramagnetic substance tagged stem cells in the subarachnoid space of the lumbar spine and some in the cauda equina. No demonstrable paramagnetic tagged stem cell was seen at the cervical spine level. (White arrow; hyposignal intensity in the subarachnoid space at lumbar spine).

**Figure 3:**
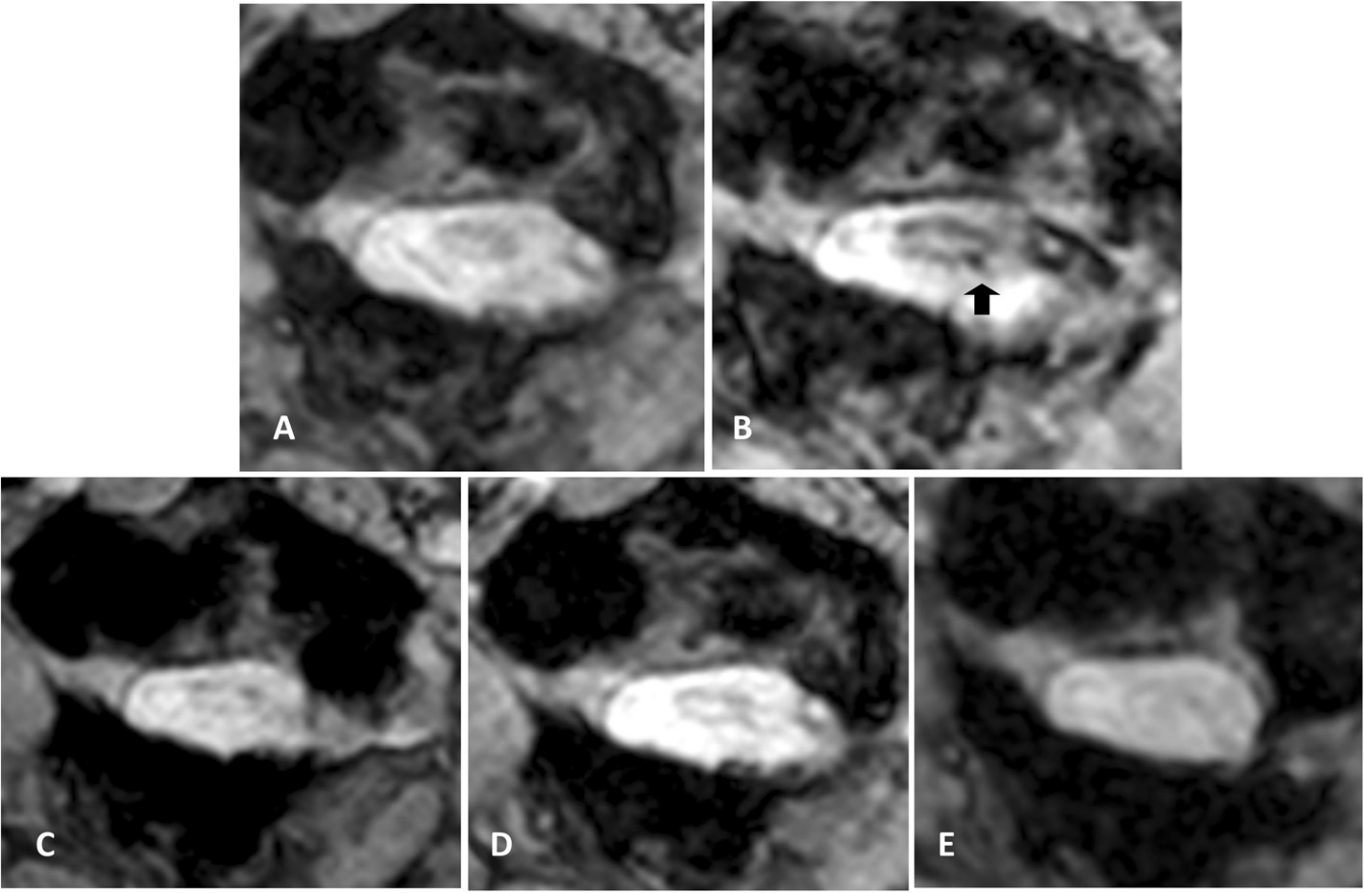
**Timeline of axial T2-star echo5 magnetic resonance imaging scans of his cervical spine at the C1 level on pre-transplantation (A), two days (B), two weeks (C), one month (D) and seven months (E) after stem cell transplantation.** The axial T2-star scan taken two days after stem cell transplantation **(B)** showed focal hyposignal intensity of tagged stem cells in the surface of the pathological upper cervical cord (arrow) at the axial T2-star scan taken two weeks after stem cell transplantation showed fading of the focal hyposignal intensity of tagged stem cells **(C)**, which disappeared on the scans taken at one and seven months.

A 3.0 Tesla MRI scan of his spine was performed immediately after stem cell transplantation. The T2-weighted scan of his whole spine and axial T2-weighted scan of his lumbar spine showed hyposignal intensity of paramagnetic substance tagged stem cells in the subarachnoid space and some in the area of the cauda equina, but no demonstrable hyposignal intensity at the cervical spine level (Figure 2). At 48 hours, there was focal hyposignal intensity of tagged stem cells at the injured cervical spinal cord; the focal hyposignal intensity of tagged stem cells faded at two weeks and had disappeared in the MRI scans taken at two months and seven months (Figure 3). There was no change of spinal cord structure in any follow-up MRI scans.

## Discussion

Intrathecal transplantation is one of the less invasive procedures for stem cell transplantation in spinal cord injury. To the best of our knowledge, this is the first clinical human case to show positive evidence of tracking stem cell migration from intrathecal transplantation at the lumbar region to the cervical region in chronic spinal cord injury. The SPION signals could be detected by serial MRI scans at the injured cervical spinal cord after intrathecal transplantation at the lumbar level.

In previous clinical studies, the intrathecal stem cell transplantation was mainly focused on clinical outcomes (motor improvement, sensory improvement or electrophysiology) [12,17,18]. There is only one report from Callera and de Melo [19] which evaluated the migration of autologous bone marrow CD34+ cells, extracellularly labeled with magnetic beads, after intrathecal transplantation. The study detected a hypointense signal at the chronic injured spinal cord but the migrated cells could not reach up to the injured cervical spinal cord [19]. However, in our case, the transplanted cells could reach up to the injured cervical spinal cord. It is possible that the setting of our patient in the Trendelenburg position for 24 hours after the transplantation allowed the cells to fall from the lumbar to the cervical level; the question of whether this is the reason for the upward migration of cells requires further studies.

Tracking cells with SPION labeling by MRI is based on the use of magnetic particles. This technique is straight forward in locating the transplanted cells [20]. The signal intensity which is detected by MRI can be readily identified and correlated with imaging anatomy; this would be an advantage for evaluation of the effectiveness of the transplantation process [21]. Nevertheless, the viability and function of BMSCs could not be evaluated with this direct cell labeling technique [22]. The hyposignal intensity from SPION labeling which is detected by MRI could not distinguish the viable cells from the dead cells or macrophages [23]. The fading of focal hyposignal intensity at the two-week follow-up MRI scan could be due to the division of viable cells or from the scavenging of dead cells by macrophages [22,23]. The fading of hyposignal intensity at the two-week follow-up MRI scan remained sufficient to track the transplanted stem cells; however, it would not be enough for tracking long-term cell survival.

There are new techniques for tracking and monitoring the viability of transplanted cells, such as reporter gene imaging, which uses specific probes to express reporter proteins from living cells. The reporter signals can be identified with many kinds of imaging, such as MRI, positron emission tomography (PET) scanning or bioluminescence imaging [23,24]. However, the concern of using reporter gene imaging in humans is mutagenesis from processes that require some transfection of genetic materials [23,24].

The neurological deficit in our patient was not significantly improved from the treatment; moreover, the unpleasant neuropathic pain increased obviously. The other complications that occurred in our patient were headache, fever and transient neurological deficit. These complications were reported in the previous literature of intrathecal stem cell transplantation, even without stem cell labeling [25-27]. There are some possible causes of the immunological reaction from this procedure. The first possibility is contamination from bovine serum in the culture process, despite multiple washing before transplantation. The second possibility is the reaction of the body toward SPIONs remnant during the transplantation procedure, or the spreading of SPIONs from apoptosis of BMSCs. Although the clinical safety of SPIONs in liver parenchymal MRI is proven, they might stimulate the immune system in the arachnoid space. Therefore, the intrathecal transplantation of stem cells should be closely observed in the first 48 hours after transplantation. The role of prophylaxis, such as steroids as a pre-medication to prevent immune reaction, may be considered in further studies.

Our case report clearly demonstrates the existence of signal intensity SPIONs at the injured site, away from the administered site. Nevertheless, the engraftment, viability and function of the cells could not be evaluated, along with the limitations of the SPIONs labeling technique.

## Conclusions

Intrathecal injection of the cells at the lumbar spine level could deliver the BMSCs to the chronic injured spinal cord at the upper cervical level. The BMSCs labeled with SPIONs could be detected by the hyposignal intensity with serial MRI. Transient complications should be observed closely in first 48 hours after transplantation. Further study should be carried out to evaluate the result of the treatment.

## Consent

Written informed consent was obtained from the patient for publication of this case report and any accompanying images. A copy of the written consent is available for review by the Editor-in-Chief of this journal.

## Abbreviations

BMSCs: Bone marrow-derived mesenchymal stem cells
CT: Computed tomography
DMEM: Dulbecco’s modified eagle’s medium
MRI: Magnetic resonance imaging
NEX: Number of excitations
SENSE: Sensitivity encoding
SPIONs: Superparamagnetic iron oxide nanoparticles
TE: The echo time
TR: The repetition time
